# 20 Years of DIEAP Flap Breast Reconstruction: A Big Data Analysis

**DOI:** 10.1038/s41598-019-49125-w

**Published:** 2019-09-09

**Authors:** Bernard Depypere, Sofie Herregods, Jacob Denolf, Louis-Philippe Kerkhove, Laurent Mainil, Tom Vyncke, Phillip Blondeel, Herman Depypere

**Affiliations:** 1 Department of Plastic & Reconstructive Surgery UZ Gent, Corneel Heymanslaan 10, Gent, Belgium; 2 Crunch Analytics, Rodelijvekensstraat 28 bus 002, Gent, Belgium; 3 Faculty of Economics and Business Administration, Gent University, Tweekerkenstraat 2, Gent, 9000 Belgium

**Keywords:** Reconstruction, Outcomes research

## Abstract

With every hospital admission, a vast amount of data is collected from every patient. Big data can help in data mining and processing of this volume of data. The goal of this study is to investigate the potential of big data analyses by analyzing clinically relevant data from the immediate postoperative phase using big data mining techniques. A second aim is to understand the importance of different postoperative parameters. We analyzed all data generated during the admission of 739 women undergoing a free DIEAP flap breast reconstruction. The patients’ complete midcare nursing report, laboratory data, operative reports and drug schedule were examined (7,405,359 data points). The duration of anesthesia does not predict the need for revision. Low Red Blood cell Counts (3.53 × 10^6^/µL versus 3.79 × 10^6^/µL, p < 0.001) and a low MAP (MAP = 73.37 versus 76.62; p < 0.001) postoperatively are correlated with significantly more revisions. Different drugs (asthma/COPD medication, Butyrophenones) can also play a significant role in the success of the free flap. In a world that is becoming more data driven, there is a clear need for electronic medical records which are easy to use for the practitioner, nursing staff, and the researcher. Very large datasets can be used, and big data analysis allows a relatively easy and fast interpretation all this information.

## Introduction

Artificial Intelligence (AI) is one of the new technological forces that will shape our future^1^. Big data and machine learning are subfields of AI. With every hospital admission, a vast amount of data is collected from every patient. This volume of data extends beyond the physician’s ability to process and is often not used to its full potential^2^. Big data can help in data mining and processing, and it can help in making predictions.

When first described in 1994, a free DIEAP flap for breast reconstruction was considered new and extravagant, but nowadays it is the gold standard in autologous breast reconstruction^3^. The DIEAP flap is considered a safe and reliable technique. Nevertheless, complications such as thrombi in the microsurgical anastomosis necessitate revision surgery as they can lead to partial or complete necrosis of the flap. This is of course disastrous for the patient and comes with additional hospital stay and costs. Big data is a new and growing trend at present. As innovators, plastic surgeons must adapt to this trend and use it to deliver more efficient health care and improve surgical outcomes^2^. The first big data analyses have been carried out^4^ and many good reviews have been written on this topic^5,6^ but they are limited to describing only preoperative conditions or postoperative results.

The goal of this study is to investigate the potential of big data analyses by analyzing clinically relevant data from the immediate postoperative phase using big data mining techniques, statistical analysis and predictive analytics. A second aim is to understand the importance of different postoperative parameters for free flap breast reconstructive surgery (e.g. MAP, blood loss and pain).

## Patients and Methods

### Study design

All research performed was in accordance with institutional guidelines and regulations. After receiving ethical board approval (UZ Gent Ethics Committee, EC2016/0881) we reviewed our electronic health records from 2004 (introduction of electronic medical records at our hospital) until 2017 for patients who underwent a DIEAP flap breast reconstruction and/or revision surgery. We created an anonymized file so no informed consent from the patients was needed for our ethical board. This was acceptable for our ethical board and we were informed on the risks of recruiting this data. We counted 965 DIEAP flaps in 739 patients. The patients’ complete midcare nursing report, laboratory data, operative reports and drug schedule were examined. Depending on the variable investigated, a different number of patients will be reported throughout the article as not all patient files were complete. As an example, half of the patients underwent their preoperative tests in our hospital, the other half with their general practitioner.

### Data collection

**Figure 1:**
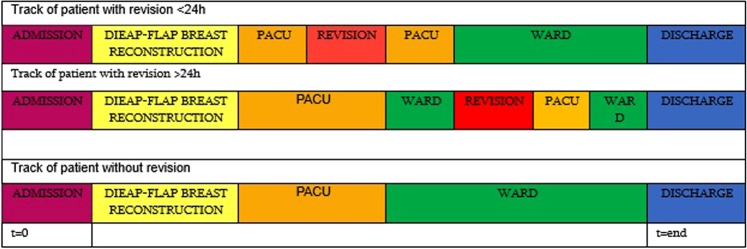
Track of patient undergoing DIEAP-flap breast reconstruction.

Raw data was obtained from the ICT department in.csv format. Every data point had a time label. We used the “RStudio project for statistical computing” software. All data was put on a timeline prior to analysis. Figure 1 shows the tracking of the patient throughout her stay. All patients are admitted the day before surgery. During surgery, intraoperative fluid administration is compared to the patient’s diuresis (1 mL/Kg/h). To maintain an adequate blood pressure, fluid challenges are given. Intraoperatively, there is no pharmacological assistance except for rare cases when phenyl epinephrine is administered when the blood pressure decreases too much. Vasopressors are rarely administered postoperatively. Intraoperative pain medication is either long or short acting opioids (sufentanil or remifentanil). Patients undergo surgery in the morning and after the procedure they are transferred to the post anesthesia care unit (PACU) where they stay overnight as the first 24 hours are critical. After inspection by the senior surgeon the next day, the patients can go to the ward. On the PACU and ward patients receive a combination of paracetamol, NSAID and tramadol. Once on the ward, a patient is rarely taken back to the operating theatre. The total dataset consisted of 7,405,359 data points which resulted in a file size of 107.4 Megabytes.

### Statistical analysis

The analysis consisted of two major parts. In the first part, information of nine different data sources was combined. Four of these data sources contained information concerning medication administration, two contained lab analysis results, one contained postoperative observations, another one contained information about the procedures themselves and a last one contained patient data. Due to the distributed nature of the relevant data and the size of the available data, automated algorithms were written to create workable basetables and to perform multiple sanity checks (e.g. only retain information on medication administrations within a sensible time period of the operation). All these manipulations were done in RStudio. This resulted in multiple basetables, each linked to a certain (set of) research question(s). The number of patients and the number of observations per patient can differ on each basetable due to inherent non-consistent rhythm of these observations, changes in observation policies in the time frame of these observations (which is close to eight years) and due to the variety in time that patients spent at the hospital. All data is included in the analysis and an incomplete dataset did not exclude the patient from the analysis. Part two consisted of performing automated statistical tests on these basetables, linked to each of the research questions. The automated nature is due to the big amount of different observations being described (e.g. 724 different unique sorts of medications being administered). These were also performed in RStudio. As two groups are being contrasted (revision versus non-revision), t-tests were used for continuous variables and chi-square tests were used for categorical variables. Postoperative observations were grouped in buckets depending on the time since the operation. We only investigated the effects of single variables without looking into possible synergistic or antagonistic effects, as we were mainly looking for indicators and predictors, not necessarily causal effects.

## Results

Variables which remained stable during admission (age, hospital stay,…) are discussed in ‘Descriptive Data’. These data do not differ from regular statistical data and analyses. Variables which fluctuated strongly during admission (pain, hemoglobin, …) were provided with a time label and the variation was measured. These calculations demand ‘Big Data’-mining techniques, statistical analysis and are described in ‘Longitudinal Analysis’.

### Descriptive data

We performed 965 DIEAP flap breast reconstructions in 739 patients with 444 unilateral and 295 bilateral cases. Within the 295 bilateral cases, 521 DIEAP flaps were harvested. If in a bilateral case a SIEAP flap or SCIP flap was harvested on one side, this side was excluded from analysis since these have smaller pedicles and have a different anatomy. The mean age of patients was 46.7 years. The difference in age was not found to be statistically significant between patients who underwent revision surgery and those who didn’t (p = 0.284). The mean duration of a unilateral DIEAP flap was 387.12 min or 6.45 h. A bilateral case took 567.27 min or 9.45 h. No statistical significance was found between cases with and without a need for revision surgery. Neither unilateral (385.17 min versus 417.19 min, p = 0.072) nor bilateral cases (564.21 min versus 605.27 min, p = 0.063) showed significantly more revisions with longer anesthesia time. From the 965 flaps, 48 needed revision surgery. As causes for revision we noted 26 venous thrombi, 10 arterial thrombi, 4 hematomas and 8 miscellaneous (in 6 cases the perforator or vein was too small to circulate the entire flap, in 2 cases the pedicle was kinked or spastic). All the flaps were used for breast reconstruction, 41.2% were primary, 47.2% secondary and 11.6% tertiary. For obvious reasons, the duration of hospital stay is significantly longer when a patient underwent revision surgery (6.79 days vs. 8.90 days, p < 0.0001). These patients needed to recuperate from the extra anesthesia, the operative procedure in general and its possible complications (Table 1).

**Table 1:** Descriptive data.

	Total Database			Split Database (no revision/revision)		
	Mean	Min	Max	Mean (no revision)	Mean (revision)	P-value
Age (years)	46.70	16	76	46.60	48.20	0.284
Hospital stay (days)	6.93	4	34	6.79	8.90	<0.0001
Duration Anesthesia Unilateral DIEAP flap (min)	387.12	195	858	385.17	417.19	0.072
Duration Anesthesia Bilateral DIEAP flap (min)	567.27	359	840	564.21	605.27	0.063
Time till revision (h)	20.73	0,25	137.67	—	—	
Weight flap (g)	809.10	193	2300	804.16	1004.60	0.239
Weight flap after shaping (g)	527.72	114	1968	525.34	565.44	0.263
Ischemia (min)	67.61	25	196	67.38	71.54	0.409

Average time till revision was 20.73 h, with the shortest being 15 min and the longest 5.74 days. Intra-operative revisions are not taken into account, although they might have led to longer anesthesia time and ischemia time.

### Longitudinal analysis

Here we discuss the time-sensitive data. Every single data point has a time label and this has two consequences: firstly, the amount of data on which we base our conclusions changes over time. Secondly, the amount of data is larger compared with a regular statistical analysis, which leads to a high power of our statistical analysis resulting in very small differences being statistically significant but not necessarily clinically relevant. In order to appreciate the outcome of every analysis, the number of data points is indicated.

#### Blood pressure

Blood pressure (BP) is monitored in every patient during surgery. Unfortunately, no data was collected during surgery because these values are noted in handwritten files. The first values after surgery are recorded on the PACU and repeated every 30 min, and on the ward every 3 h. Systolic and diastolic pressures are converted to Mean Arterial Pressure (MAP), which is considered to be the perfusion pressure of the flap. Only values from patients without revision or, in case of revision, patients before their revision are included to emphasize the predictive value of the MAP. Values of patients after revision are not included. Between the 3^rd^ and 6^th^ hour postoperatively, 2117 data points were collected from 545 patients. Between the 21^th^ and 24^th^ hour, 578 data points from 512 patients were collected. A total of 6726 blood pressures were observed.

**Figure 2:**
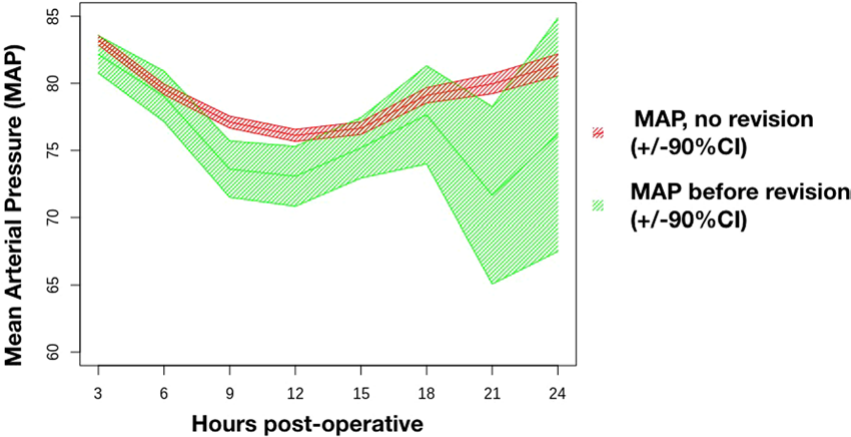
Mean Arterial Pressure postoperatively (+/− 90% CI).

In the immediate postoperative setting, we note a similar drop in MAP in all patients. In patients needing revision surgery, we notice that the MAP drops significantly more between 6 and 12 hours postoperatively from their breast reconstruction (MAP = 73.37 versus 76.62; p < 0.001) (Fig. 2).

#### Pain score

**Figure 3:**
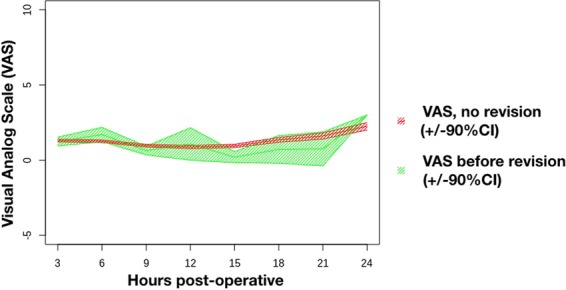
Postoperative VAS-score in rest (+/−90% CI).

Postoperative pain is measured with the Visual Analog Scale (VAS)-score. Data points range from 752 measurements at 3 h to 129 at 21 h after surgery. A marginally statistically significant difference was found between the 2 groups (p = 0.03). During their stay, patients who underwent revision surgery experienced more pain than patients with an uneventful recovery (VAS = 2.98 versus 2.75 p < 0.001) (Data not shown). Unfortunately, postoperative pain after breast reconstruction is a bad predictor for revision as there was no difference in the first 24 hours which are most critical (Fig. 3).

#### Blood loss

**Figure 4:**
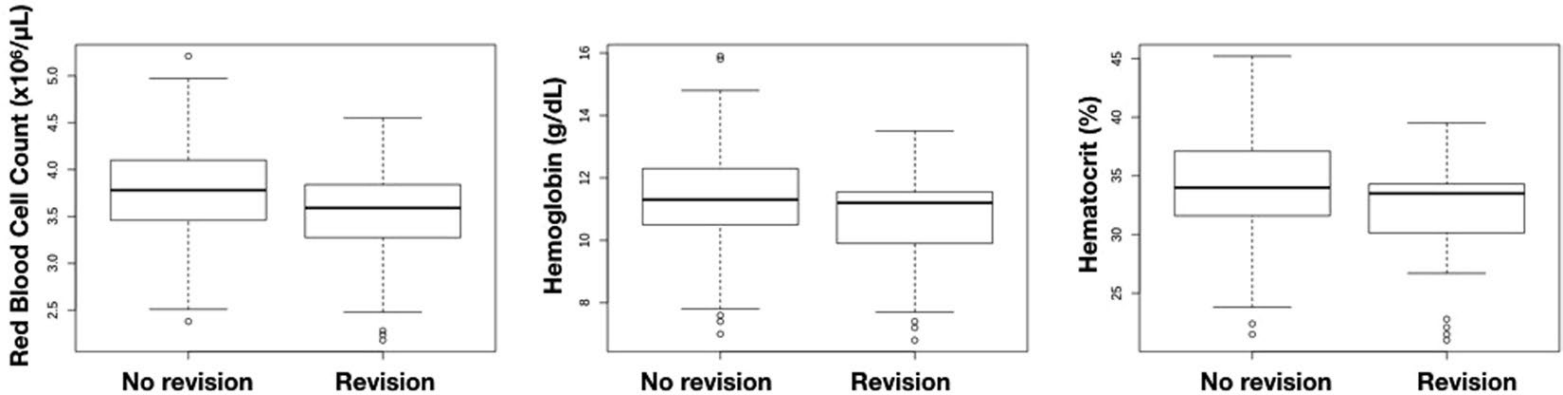
Postoperative blood work. Left RBC, middle Hemoglobin, right Hematocrit.

For every patient, the Red Blood cell Count (RBC), hemoglobin (HGB) and hematocrit (HCT) are checked among other parameters. Respectively 1915, 2237 and 2019 lab values were analyzed. In a total of 527 patients (44 revision cases and 483 uneventful cases) we noted significantly lower RBC in the revision group (3.53 × 10^6^/µL versus 3.79 × 10^6^/µL, p < 0.001). Postoperative Hemoglobin was measured in 538 patients and averaged out at 11.41 g/dL in the non-revision group compared to 10.72 g/dL in the revision group (p = 0.0018). In a total of 532 patients, the postoperative Hematocrit was 34.21% in the non-revision group compared to 32.23% in the revision group (p = 0.0019) (Fig. 4). Blood loss is a good predictive parameter. To emphasize the predictive value, a post-hoc analysis of the RBC, HGB and HCT values was done from patients *before* they underwent revision surgery (32 revision cases and 483 uneventful cases). This showed that they already had significantly lower values immediately after their DIEAP flap procedure (RBC 3.38 × 10^6^/µL versus 3.51 × 10^6^/µL, p = 0.002), (HGB 10.26 g/dL versus 10.71 g/dL, p = 0.005), (HCT 31.27% versus 32.22% p < 0.008).

**Figure 5:**
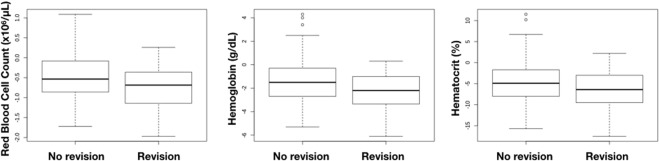
Blood loss after DIEAP flap without and with revision surgery.

More interesting are the results obtained from a smaller group of patients who did their preoperative blood work in our hospital. This allows us to estimate the intrapatient blood loss after DIEAP flap breast reconstruction without a need for revision and compare it to the blood loss after DIEAP flap followed by revision surgery. There were no preoperative differences in RBC, hemoglobin or hematocrit (data not shown). In a total of 235 patients, RBC after surgery dropped with 0.48 × 10^6^/µL in the non-revision group compared to a drop of 0.74 × 10^6^/µL in the revision group (p = 0.035). Comparable results were obtained for hemoglobin [−1.45 g/dL (no revision) versus −2.33 g/dL (revision), n = 253; p = 0.017] and hematocrit [−4.51% (no revision) versus −6.63% (revision), n = 246; p = 0.047] (Fig. 5).

#### Medication

Complete medication schedules were obtained from 472 patients (796 different drugs and 85,641 drug administrations to patients). The extensive medication list was categorized and an analysis was carried out based on these categories. Chronic medications are listed under “Home medication” and new medications under “Medication started in hospital” (Table 2).

**Table 2:** Analysis of medication. A bold font signifies a p-value < 0.05.

Home medications	No revision (n = 434)	Revision (n = 38)	p-value
antidepressiva	28	3	0.9977
antidiabetica	9	1	1
anti-epileptica	1	0	1
antihistaminica	12	2	0.71
antihypercholesterolemica	23	3	0.7629
antihypertensiva	64	8	0.4229
antipsychotica	6	1	1
aromatase inhibitor	9	1	1
benzodiazepine	220	24	0.1918
corticosteroids	384	35	0.6811
COPD/Asthma medication	19	6	0.0084
triptans	1	0	1
thyroxine	4	1	0.7595
vitamines/minerals	129	15	0.2855
**Medication started in hospital**	**No revision (n** = **434)**	**Revision (n** = **38)**	**p-value**
antibiotic	416	37	0.9796
antinausea	396	36	0.6617
antiobstipation	378	32	0.799
antireflux	396	35	1
antithrombosis	416	37	0.9796
Leeches	0	4	<0.001
painkillers	434	38	1
phenylephrine	2	2	0.0297
dobutamine	0	3	<0.001
butyrofenon (droperidol & haldol)	105	17	0.0099

## Discussion

Many good reports have been written on autologous breast reconstruction, and its safety has been proven^4,7–10^. As a novelty we would like to indicate the potential impact of Data Mining in future practice. Our “descriptive data” doesn’t bring any new insights and only confirms findings of previous authors. Our patients have the same age and both the number of perforators and length of hospital stay are comparable^8^. Increased duration of anesthesia did not predict flap failure^11^. In contrast, new insights become clear when looking at the “Longitudinal Analysis”. Mean arterial pressure (MAP) was investigated by Nelson *et al*. and post-hoc analysis couldn’t show any significant difference in patients with and without thrombotic events (p = 0.17)^12^. Our initial analysis of the MAP did not show any significant difference either (data not shown). Only when we put a time label on every measurement, we could ascertain that there is in fact a significant difference in MAP between our two groups, but only between 6 and 12 hours postoperatively. This can be an important item for future postoperative management. This should also be one of the variables to include if we would ever make a predictive model in the future. Patients wake up after surgery with initial pain and anxiety during extubation which explains the higher MAP. The MAP drops during the first hours on the PACU, when patients find rest and when their pain is under control. Their MAP then increases slowly when then women become more alert 12 hours after surgery. The MAP gives us an estimate of the blood pressure in our microvascular anastomosis. A lower MAP signals a lower flow, which in turn is more thrombogenic.

Blood loss is also well documented^10,12^. As every lab result has a time label, we could generate the data of individual patients and compare their pre- and postoperative values. This allowed us to draw two conclusions. Firstly, revision patients had a significantly lower RBC, HGB and HCT values after the initial procedure, compared to non-revision patients. Secondly, a comparison of the intrapatient values allowed us to calculate the average blood loss in an autologous breast reconstruction with a DIEAP flap. In the non-revision group, the RBC dropped by 0.48 × 10^6^/µL, Hemoglobin dropped by 1.45 g/dL and Hematocrit dropped by 4.51%. The decreases in the revision group were: RBC −0.74 × 10^6^/µL (p = 0.035), Hemoglobin −2.33 g/dL (p = 0.017) and Hematocrit −6.63% (p = 0.047). In other words, a higher blood loss can predict the need for revision surgery.

Medication analysis was performed since this data was available in large amounts (796 different drugs and 85,641 drug administrations). We are aware of the difficulty to establish a causal link between an administered drug and revision surgery. At least we hope to demonstrate the safety of some commonly used medications (antidepressiva, anti-histaminica, benzodiazipines, thyroxine). Obviously, drugs (such as phenylephrine, dobutamine, leeches) that are given when a free flap is not doing well, are not a cause of revision. COPD patients are known to have higher surgical complication rates^8^. In our analysis, a statistically significant correlation was found between COPD/asthma medication (see Tables 2, 3) and the need for revision surgery. This means that COPD patients, apart from having more major delayed complications as stated by Fisher *et al*., also have a higher risk for thrombotic events in their anastomosis.

**Table 3:** List of medications included in the category “COPD/Asthma medication”.

Name	Mechanism
**COPD/Asthma medication category**	
Acetylcysteine	Mucolytica
Dextromethorphan	Antitussiva
Ipratropium + fenoterol	Anticholinergicum + Β_2_-mimeticum
Fluticasone, Budesonide, Beclomethasone	Inhalation corticosteroid
Montelukast	Leukotriene receptor antagonist
Mometasone	Corticosteroid
Salmeterol + fluticason	Β_2_-mimeticum + Inhalation corticosteroid
Ipratropium	Anticholinergicum
Doxapram	Analepticum/Respiratory stimulant

Haldol and Droperidol are two drugs administered in our hospital in the immediate postoperative setting, against delirium and nausea. These Butyrophenones are dopamine D2-receptor antagonists and are classified as highly potent neuroleptics. The anti-dopaminergic effects at the Chemoreceptor Trigger Zone (CTZ) accounts for the strong antiemetic activity. Known peripheral effects include: relaxation of the gastric sphincter muscle, anticholinergic side effects, prolongation of the QT-interval, dysrhythmias and depression of the respiratory system^13^. Seemingly women who needed revision surgery took more Butyrophenones. Knowing these women had 2 surgeries in a short period of time, nausea and delirium might be higher in this group. Whether these drugs are a causal factor for the need for revision is not clear and more research is necessary to distinguish effect from cause.

### Limitations

Our research has several limitations. First and foremost, in big analyses like ours, there will always be missing data. Data is entered wrongly or in different tab sheets. This makes sanity checks extremely important. Secondly, our dataset includes 965 DIEAP-flaps and only 48 revisions (or 4.97%) which limits the power of our statistical analysis. One solution for this problem could be a multicenter study. Thirdly and maybe the most important factor, a correlation does not mean there is a causation. Therefore, we have to be precautious in drawing conclusions. Thirdly, no intra-operative data was available since this is still registered on paper files in our institution.

## Conclusion

Every reconstructive surgeon performing free flaps tries to limit his or her revision rate in every way possible and every single one of them also knows that there are numerous possible causes leading to revision. This cause can be either mechanic (wrong pedicle, poor anastomosis technique,…) or intrinsic (bad perfusion, bad coagulation, comorbidities,…). In a data analysis, the distinction between the two is not visible and for that reason we should always be cautious in drawing conclusions. We can conclude that anesthesia time does not predict the need for revision surgery. Average blood loss in DIEAP flap breast reconstruction is 0.48 × 10^6^/µL RBC. Average blood loss in DIEAP flap breast reconstruction followed by revision is 0.74 × 10^6^/µL RBC. Results are comparable for Hemoglobin and Hematocrit. A lower MAP is correlated with higher need for revision surgery. We recommend a postoperative MAP of at least 77 mmHg. Apart from blood pressure, blood oxygenation may also play a significant role in the success of the free flap, since we saw higher revision rates in patients who took COPD/Asthma medication and patients who took Butyrophenones, which are known to cause sedation and depress the respiratory system.

In a world that is becoming more data driven, there is a clear need for electronic medical records which are easy to use for the practitioner and nursing staff, as well as for the researcher in need of correct and complete data. In the past datasets were made by people extracting data from (written) patient records. In the future, datasets will be easier to generate as it will be simple to extract them from the electronic medical records. Having a computer generate your dataset (like ours) only requires testing your data thoroughly, hence avoiding duplicates and wrongly entered data.

Machine Learning (ML) is a subset of AI. It is the engine of an AI-device making its own decisions. If ML is considered to be the engine, then the data can be considered the fuel. Even though our dataset was generated by Data Mining, over 7 million data points is still too little to educate a machine and make predictions. A future project would include a multicenter study where we can combine all the data. Then we might be able to create an AI-device capable of telling us if a free flap will need revision before it becomes clinically noticeable. Until that day we should get acquainted with the techniques of Data Mining and Big Data. It is a different kind of statistics and a different way of looking at the postoperative results.
