# Author Correction: 20 Years of DIEAP Flap Breast Reconstruction: A Big Data Analysis

**DOI:** 10.1038/s41598-020-58292-0

**Published:** 2020-01-24

**Authors:** Bernard Depypere, Sofie Herregods, Jacob Denolf, Louis-Philippe Kerkhove, Laurent Mainil, Tom Vyncke, Phillip Blondeel, Herman Depypere

**Affiliations:** 1 Department of Plastic & Reconstructive Surgery UZ Gent, Corneel Heymanslaan 10, Gent, Belgium; 2 Crunch Analytics, Rodelijvekensstraat 28 bus 002, Gent, Belgium; 3 Faculty of Economics and Business Administration, Gent University, Tweekerkenstraat 2, Gent, 9000 Belgium

Correction to: *Scientific Reports* 10.1038/s41598-019-49125-w, published online 09 September 2019

The original version of this Article omitted an affiliation for Louis-Philippe Kerkhove. The correct affiliations for Louis-Philippe Kerkhove are listed below:

Crunch Analytics, Rodelijvekensstraat 28 bus 002, Gent, Belgium

Faculty of Economics and Business Administration, Gent University, Tweekerkenstraat 2, Gent 9000, Belgium

This has now been corrected in the HTML and PDF versions of this Article.

